# Restricted Self-Transition: A Journey of Divorcees Through Lasting Marital Dissolution in Eastern European Society

**DOI:** 10.5964/ejop.9619

**Published:** 2023-08-31

**Authors:** Lina Butkutė, Dimitri Mortelmans, Jolanta Sondaitė

**Affiliations:** 1 *Faculty of Social and Human Studies, Mykolas Romeris University, Vilnius, Lithuania*; 2 *Faculty of Social Sciences, University of Antwerp, Antwerp, Belgium*; Université de Fribourg, Fribourg, Switzerland

**Keywords:** self, lasting divorce, qualitative study, Lithuania

## Abstract

Although most empirical research has focused on divorcing individuals’ experiences before or after marriage dissolution, how people understand and evaluate themselves during their lasting divorce processes has been largely understudied. We aimed to close this gap by learning how individuals regard their longer-lasting divorce process and how those experiences could relate to changes of self. Data were collected via semi-structured interviews and then analyzed by applying a grounded theory approach. Twenty-one research participants residing in Lithuania who were 6 months or more into their divorce processes (not living together or in a litigation process) participated in the study. By allowing participants to reflect on their ongoing divorce, data indicated three main categories illuminating the changes in self: temporal self-disruption, restricted self-transition, and transition-supporting strategies. These interconnected categories point toward complex paths of the divorcees from experienced losses toward a more stable and clear yet not finalized self-redefinition. Individuals’ increased vulnerability, especially during the first years of the divorce, requires particular attention from child protection officers, lawyers, mediators, and other involved professionals. Unfortunately, support is often unavailable or refuted due to the perceived low effectiveness and lack of professionalism.

Divorce is not a single, discrete stressful event. Instead, the most commonly accepted theoretical divorce model supports a process perspective (Amato, 2010). Most couples find ways to manage timely their marriage’s formal termination; however, up to 25% result in long-lasting, complex, usually highly conflictual, and more expensive trajectories (Smyth & Moloney, 2017). Breaking marital bonds with significant others usually associates with many negative consequences for divorcing individuals, including increased unclarity and instability of self-perception and decreased general well-being (Schiller et al., 2016; Slotter et al., 2014). The above negative consequences could lead to an expected prolonged (legal) divorce trajectory associated with more pronounced and complex inner changes. However, some research indicates that neither conflict nor duration of divorce relates to depressive feelings or life satisfaction (Symoens et al., 2013). A significant number of divorcees achieve observed gains in perceptions about themselves and psychological well-being (Treloar, 2019).

Despite the importance of the topic and several studies tackling the divorce phenomenon, in their recent literature overview, Haddad et al. (2016) argue that many of these studies provide only theoretical knowledge and backgrounds of ongoing divorce. Data are primarily gathered in pre-divorce or post-divorce settings (Crabtree & Harris, 2020; Francia et al., 2019). Most of the studies on this topic employ quantitative methods that focus on variables the researchers identified and therefore, are unlikely to represent divorcees' contextualized experiences (Mercer, 2011). One of a few qualitative studies examining the ongoing legal divorce period focused primarily on the conflict tactics between divorcing partners, as the researchers observed (see Gulbrandsen et al., 2018). However, their results did not include divorcees' perspectives. Individuals' views of themselves amidst their divorce and the associated emotions are important when discussing timely and more effective divorce conflict-resolution approaches (Hopper, 2001; Slotter & Walsh, 2017).

To respond to the above gaps and conflicting results, with this study, we aim to show what transformations of the self individuals experience during their lasting divorce process and the ways they respond to them to make divorce life more bearable. We advance the divorce literature in several ways. First, taking the lasting divorce process framework as a focal point prompts looking at divorce as a more complex process, exploring how various transitions related to a divorce account for variance in outcomes (Amato, 2010; Crabtree & Harris, 2020). Second, as divorce-related self-perceptions diminish over time (Booth & Amato, 1991; Strohschein, 2005), providing real-time data permits contextualizing lived experiences and provides a better understanding of the phenomenon. Third, grounding results in the divorcees’ standpoint allows accommodating the complexity and variation of the self in the specific context, opposite to generalized models of a “theoretical divorcee” (Bertelsen, 2023; Mercer, 2011). Finally, we present three main categories, providing an in-depth understanding of the transformations and preservation of the self. More profound empirical knowledge of the phenomenon is needed to ensure that the desired divorce-related results are more durable and that they are achieved more effectively (Bartolucci & Gallo, 2010; Hald et al., 2020).

## Literature Review

People create themselves by fusing with various elements from valued life domains (Tabri et al., 2017). For example, the more the person values particular self-aspects related to the relationship, the more aspects of it define that person (Tabri et al., 2017). People strive to maintain stability through time and place, and they are usually reluctant to change (Lodi-Smith et al., 2017). Stability and clarity of the self-construal associate with low levels of distress, high self-esteem, active coping styles, and low neuroticism levels (Smith et al., 1996). However, divorce often stands in the way of individuals’ ability to narrate themselves and it leads to disturbances in inner clarity (Schiller et al., 2016). Experienced disturbances during life transitions can catalyze the adjustment and emergence of new forms of conduct, which in some cases might involve restructuring the whole self system (Zittoun, 2008).

The self can be understood as an intra-psychological structure concerned with one’s self-perception that evaluative inferences continually reinforce, which reflect both cognitive and affective responses (Arens et al., 2011; Kohut, 2013). The experience of the self includes consciousness of one’s physicality as well as one’s inner character and emotional life. Mattingly et al. (2014) underlined the above duality of the self when discussing changes related to romantic relationship dissolutions. They argued that the self changes along two dimensions: diversity (increased or decreased) and experienced emotional response (positivity or negativity) regarding the change. The more the person values particular self-aspects related to marriage, the more aspects of it define that person (Tabri et al., 2017). In that case, losing valued self-aspects is particularly difficult because doing so feels like losing significant parts of oneself. However, in some relationships, individuals might experience decreased positive self-attributes or increased negative ones, for example, by developing new bad habits. Consequently, such a bond’s dissolution would provide a sense of relief when it ended, allowing the rediscovery of neglected self-aspects and the experience of growth (Lewandowski & Bizzoco, 2007).

Literature indicates that self-transformation is tightly connected to changes related to the roles established during marriage. Individuals stand before a task to redefine their identity to make it distinct from the identity of both the former couple and the former spouse (Bohannan, 1971). In his qualitative study, Hopper (2001) conceptualized that divorce often became more than a loss of a partner and therefore, it became more about the loss of a dream of togetherness and self-wholeness projected into the future. Particularly Lithuanians still consider marriage as a highly valued, sacred union intended to last until death (Kanopienė et al., 2015). Breaking this sacred union brings to the foreground many internal moral disputes. Divorce also involves changes in the parental role to establish a new parental identity. This task requires transforming the emotional attachment between the parents to a functional attachment that confines the relationships to parenting alone (Emery, 2011).

Staying for a prolonged time in an unclear situation could prevent timely and effective self-redefinition and it could lead to increased inner unclarity. Qualitative research exploring the lasting separation without clear intent to divorce indicated that finding the self in lasting and unclear settings related to higher stress levels and a greater role of unclarity (Crabtree & Harris, 2020). Individuals were unsure about what was expected from them and how long such settings would last. Some researchers related experienced uncertainty about the expectations associated with particular roles and the existence of certain relational status to the so-called role-relational ambiguity phenomenon (Tabor, 2019). This phenomenon points to the unclarity of the self in ambiguous and unfinalized life situations, such as a lasting divorce process.

Individuals use various resources to help ease divorce-related stressors, among them the ones residing within individuals (e.g., coping skills, social skills) (Amato, 2000). Zittoun et al. (2003) referred to them as symbolic resources that helped to reframe and reorganize a changing situation’s uncertainty. Hattie (2008) argued that these resources or strategies were integral components of the self and they strengthened it. Therefore, to understand how the self works and on which rationales it is formed, the focus should lie on these dynamic internal strategies and choices. People mainly choose strategies to protect, preserve, and promote their selves (Alicke & Sedikides, 2009; Hepper et al., 2010). So-called self-enhancement and self-protection strategies help to preserve and enhance positively the valued self-dimensions (Sherman & Hartson, 2011). For example, viewing perceived gains and feeling positivity from otherwise adverse events relate to a smoother reconstruction of identity and increased clarity (Jayawickreme et al., 2021). Acceptance-based strategies could also be ways to support divorce transitions. These strategies focus on increasing the ability to tolerate actively and accept aversive internal experiences and to lead to further adaptive behavior (Forman & Butryn, 2015; Veletsianos et al., 2018). Last, forming a new stable romantic relationship supports the divorce adjustment process and is one of the essential factors in adjusting to post-divorce stress and self-redefinition (Langlais et al., 2016).

## Method

### Study Design

We chose a qualitative approach to investigate changes related to the self for several reasons. First, given the absence of studies investigating the lasting divorce process, a more exploratory study seemed appropriate to provide a potentially more profound understanding of the lived phenomenon among a specific group of divorcees. Second, we employed a constructivist grounded theory (CGT) because it provides a systematic approach to understand the development of the self during ongoing traumatic life events (Thornberg & Charmaz, 2014). The CGT is particularly suitable for an exploratory study such as this one because it allows remaining close to the data and contextualizing the divorcees’ experiences without preconceived or anticipated patterns. The CGT suggests that data are constructed through an ongoing interaction between participants and the researcher and that the analysis reflects both perspectives (Thornberg & Charmaz, 2014). In conducting our analysis, no explicit use was made of theoretical constructs; however, the researchers’ conceptual framework inevitably informed the analysis, as did the reflections on the findings in the discussion section. Third, we have selected the self as this study’s focus because it is a self-related construct that includes both an affective and a cognitive dimension. The self can be sufficiently broadly defined to capture a wider, more holistic set of beliefs, feelings, and used strategies associated with lasting divorce and more general life context. This study is part of the Ph.D. research trajectory. The university research ethics committee granted ethics approval (Protocol No. 6/-2021). The first author (a doctoral psychology student) carried out all data collection and led the analysis.

### Participants

The sample consisted of 21 married individuals (5 males and 16 females) who, at the time of the interviews, identified as (a) still legally married, (b) living in Lithuania, and (c) in the process of divorce for at least 6 months (not living together or in a litigation process) (Mortelmans, 2020). Participants ranged from 28 to 64 years (*M* = 43.8; *SD* = 8.24). Since the beginning of a divorce, the mean number of years was 2.1, ranging from 6 months to 4 years (*SD* = 1.11). Our participants had been married for 16.1 years on average, with a range of 2 to 40 years (*SD* = 10.63). All participants had one or more children with their divorcing spouses and identified themselves as white. All of the relationships with spouses described in this study were heterosexual and gender normative. Because the initial strategy to interview both individuals from a divorcing couple did not materialize due to the sensitivity of the divorce situation, we focused our data collection and analysis strategy on individuals instead. Consequently, 2 participants were (former) spouses of 2 other participants. Furthermore, as interviews took place online, it allowed for recruiting divorcees from various towns across the country, including cities and rural villages.

### Recruitment

We recruited the sample through convenience, snowball, and purposive sampling techniques. First, we contacted via email various social and mental health support centers, community agencies, governmental institutions, lawyers, and counselors with a request to support the recruitment process. The emailed text explained the study’s design and purpose. Second, to increase the number of participants, we utilized announcements on social media platforms, particularly Facebook, and specific divorce-related Facebook discussion groups. Third, we approached individuals from a personal network with a request to disseminate information about the research to people they knew who were going through a divorce or could share the information with others. Finally, after each interview, we asked participants to share information about the research with other individuals who fit the criteria.

### Interview Procedures

The first author contacted interested participants to arrange a 60- to 90-min. semi-structured interview by audio (*n* = 19), or video call (*n* = 2), whichever the participant preferred. Before conducting interviews, we extensively discussed the study’s goal and aim and the interview process with each participant. We underlined, among others, the anonymous and confidential handling of data, the participants’ rights to terminate the interview, and participation in the study. We conducted interviews using a topic guide developed after the initial literature review. Interview questions concerned experiences regarding the ongoing divorce process, how divorcees viewed themselves, and their interactions with other people. All interviews were audio-recorded and transcribed verbatim.

### Coding and Analysis

**Figure 1:**
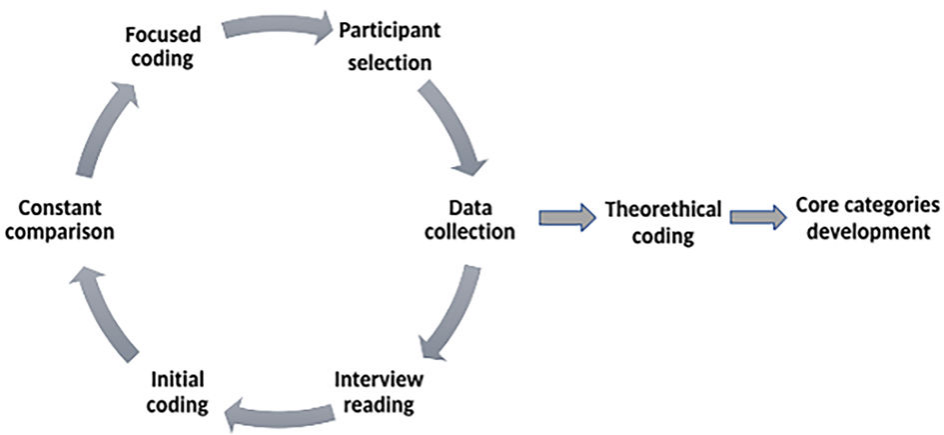
Visual Representation of the Research Data Analysis Process *Note.* Steps that were taken to arrive at the study’s core categories.

After the data were transcribed, we analyzed the data, applying a grounded theory (GT) approach informed by Charmaz (2014). Coding is essential in GT studies and occurs in particular stages, which we have followed (see Figure 1). First, the researcher read each interview to gain a holistic sense of the text and wrote a summary statement to capture the overall essence of that participant's experience. Second, readings helped identify and code what individual sentences or sentence clusters revealed about the ongoing divorce experience (initial coding). Third, by constantly comparing codes with codes and seeing how the codes coalesced, she identified the most important codes and engaged in focused coding. This coding process involves identifying the most frequently occurring and more important initial codes and using them to group data for deeper analysis. Fourth, codes were arranged in various levels of abstraction, thus creating categories. Finally, theoretical arrangements were assigned to the data and subsequently revised until the collection of structural experiences captured the similarities across and variations within the participants' experiences (theoretical coding). Although the GT name suggests that a fully fledged theory must arise from a GT study, scholars argue that significant progress toward constructing categories and spelling out links between them, with the aim of achieving conceptual clarity, is a sufficient (if not necessarily the ideal) outcome (Timonen et al., 2018). Consequently, with our study, we focused on developing core categories and the interlinkage between them to show in-depth the changing self during the lasting divorce process. Coding was done using NVivo 12 software.

### Strategies for Trustworthiness

We followed trustworthiness criteria to ensure the findings’ quality (Schwandt et al., 2007). To increase credibility, which included showing how well the findings reflect collected data (McGinley et al., 2021), we met monthly to discuss the interviews and concepts that emerged from the data and to explicate personal assumptions and biases as they surfaced. In addition, the first author practiced ongoing reflexivity to enhance confirmability, which concerned minimizing the researcher's bias (Charmaz & Thornberg, 2021). For that, she used a reflective journal. In qualitative studies, the journal records complete and thorough information (memos) about what, how, and why actions were undertaken and what conclusions we came to (and why) (Charmaz, 2014; Clarke & Braun, 2013). In memos, the first author reflected, among other considerations, on the process and practice of recruitment and data collection, analytic insights during data analysis, and emotional aspects of the research that affected the researcher and the research. The first author made memos available to two scholars who were not involved in the study but offered content and methodological expertise.

## Results

Looking at the data gathered during the interviews, main categories of understanding and evaluating self during divorce came forward: temporal self-disruption, transitional self-redefinition, and transition-supporting strategies, which are discussed below in detail.

### Temporal Self-Disruption

This concept includes two components, which indicate a kind of cracking and gap in the experience of self and emotions associated with the experience. These components are mainly focused on the disruptions in the past self as well as continuing threats to the current self.

#### Losing a Togetherness Dream

Most participants experienced their divorces as the end of the world or the dream of a sacred union “till death sets us apart.” People talked extensively about realizing that, “everything in life is falling apart” and “all dreams are collapsing,” particularly in the first year of the divorce. Emotional disconnection was one of the most crucial and complicated parts of the divorce process experienced. Katrina (37) revealed that, “emotional disconnection was very hard…. I understood how much I am glued to him. I compare it with a co-dependence on a person.” For most, this process started a long time ago. However, some argued they still found it difficult to relinquish their spouses. Consequently, forming a functional relationship with their former spouses came with its own challenges. Many mothers living with their offspring embraced the idea of co-parenting, yet they feared that disagreements regarding children would continue beyond divorce. Although for most of the divorcees, the physical separation was no longer an acute question, unresolved questions regarding the division of the property were lagging and creating future-related unclarities.

#### Losing Clarity of a Traditional Parent

Throughout the interviews, parents with minor children have underlined a need to “be a good parent” and do everything for their children’s sake. However, their ability to enact a parental role depended on whether children were living with them or their visitation was blocked in specific ways. The most challenging experiences were among parents whose children showed reluctance to interact. Those individuals were the most unsure about their parental roles. Their mixed emotions ranged from sadness about the loss of their children to anger toward their ex-spouse for manipulating them, as well as shame for, “being the mother whose children refuse to see her.” Divorcees expressed inner doubts about their importance as parents after the divorce. Paul, a father of two, doubted, “whether I will be needed [for the] children or [if it is] only the money when they are in need.” Being in increased uncertainty for a prolonged period, the doubts reached such extremes that some were elaborating to renounce officially their roles as parents. In contrast, parents residing with their children underlined the burdensome, lonely responsibility of raising their offspring without a spouse’s support. Many of them reported identifying “more with a role of a mother than a woman” as they expected to live the “intensive life of a mother in the coming years.”

### Restricted Self-Transition

This concept refers to the emerging renewed self in the midst of the ongoing divorce, paired with multiple sources preventing the finalization of the process. It is a transitional space within an increasingly unstable situation with a hope to enter potentially the new and different life conditions.

#### Re-Building Valued-Self

Besides reflecting on their losses, participants contemplated the increase in their self-worth, self-respect, and self-love. Especially divorcees who were more than a year into their divorce discussed inner healing and re-building of an “old” valued self. Individuals felt more stable, peaceful, and stronger. They set self-protecting boundaries and refused self-guilt. One participant reflected on the process as “a return to self:” “It seems like now I'm a little stronger, more stable…. Now somehow it's a lot better, a lot. There has happened a return to myself” (Daisy, 47, 3 years into divorce). With the return to self, individuals experience a sense of liberation, freeing the self from the dysfunctional family environment, and allowing the self to do things that matter most. New partners played a significant role in restoring a valued self. Participants felt loved and secure with a stronger sense of self-belief and self-worth.

#### Constrained by Uncertainty

Many individuals experienced the binding of the status of still being legally married as constraining and prohibiting from moving forward. Virginia (39), 4 years into divorce, shared her experiences of this, “You understand that you are like a free person, we do not live together for five years. But at the same time, I am still formally a married woman…it’s a complicated thing.” Divorcees talked extensively about the sense of feeling stuck in the process. They viewed divorce as something they were dragging behind that did not allow them to be free completely or to plan according to their wishes. People did not know when their divorce would be finalized and what would be the results. Divorcees saw themselves as caught in the process without a clear future, expecting the unexpected and unsure if they would be prepared to face it. Peter, a 46-year-old father, expressed the experience as finding self in the absurdity as it is in the Kafka novel, “Something will happen again, that child is again incited, again. I always get something. And you continuously feel hung, not released…. So you sit, and you wait.” This kept divorcing individuals in constant alertness and having an inability to complete the separation process.

#### Enduring Externally Derived Attacks

Many interviewees reported constant alertness due to feeling their former spouses or legal advisors were attacking or blaming them. Some of them referred to the situation as fighting in a war. The prevalence of this aspect could be partly related to the specificity of a purposefully selected sample, which includes individuals in highly conflicted litigation processes. Participants saw themselves manipulated and put down to “simply see me suffering.” They also faced direct negative labeling from their spouses. Steven, 45 and 3 years into divorce, told us, “Those night messages-letters as I call them. The topic is practically the same that you are guilty guilty guilty, a scoundrel scoundrel, but you still have a chance to improve.” Being blamed and attacked was often associated with fear and anxiety, as well as anger because most of the time, the accusations were seen as lies or tools of manipulation. Participants felt attacked not only from their spouses and legal advisors but also various employees of institutions. They felt being looked upon as fools: “those people with problems” or “a criminal caught committing some kind of terrible crime.” Negative self-attributions arose from being involved in the institutionalized divorce process as such. Experiences with various institutions (courts, social services, child protection offices, and judicial offices) left people feeling humiliated, asocial, and helpless. They saw themselves as “forgotten pieces” in the “lion’s jaws” or in “a self-moving machine” that did not care about them as human beings, their needs, feelings, and experiences.

*I feel like some kind of asocial. All these lawsuits, those things, oh Jesus, you know. I tell you, I feel like on those shows on* LNK, TV3, *or* TV Help *[reality shows], where they are showing those. I feel that level of a person. It is so low for me. (Karen, 47)*

### Transition-Supporting Strategies

This concept includes strategies that are intentionally or unintentionally used to reconstruct the self that has been disrupted with the start and continuation of the divorce. It is also associated with preserving the emerged valued self-parts in the transitional period and protecting them from possible disruptions.

#### Staying Strong and Truthful

During the interviews, participants reported two main strategies for encouraging themselves. First, divorcees reported seeing themselves as vital individuals. Experiencing the self as strong often connected to being able to provide for the new family financially, reaching professional self-realization, and having their colleagues like them. For some, the proof of their strength was a decision to divorce. As Katrina (47) stated, “Divorce is not an easy thing. It is a myth that only weak people divorce. Because being in a destructive relationship, I think, is even easier than getting out of it.” To encourage themselves, individuals talked about their determination to stand for what they valued. They reflected on having the strength to fight for what they thought was rightfully theirs and they were ready to face any obstacles to achieve it. For some, the strength to fight came from seeing themselves as truthful individuals, not having anything of which to be ashamed or for which to be blamed.

#### Counteracting the Blame

Due to the ongoing uncertainty, perceived manipulation, and humiliation, people saw themselves as “guilty without proven guilt.” These experiences enhanced a need to protect themselves by proving that reality was not how the outside world depicted it. Individuals turned toward common truths about the normality of the behavior. They discussed behaving according to certain standards applicable to the general public or specific professions. Martin (43) expressed his argumentation regarding his wife’s accusations, “She [wife] repeats [to everybody] that I scream, that I raise my voice. But it is natural, because I am a guide, for me to talk in front of an audience of 40 people.” Second, individuals used other people to prove their truthfulness. These were relatives, friends, close people, or professionals from various institutions (particularly psychologists, doctors, and private attorneys). Finally, some interviewees went a step further, pointing out their spouses’ inadequacy, calling them stupid, narcissistic, pathological liars, manipulators, and the like.

#### Reconstructing the Past

The interviews revealed that divorcees engaged in two types of past reconstruction. From one side, it is related to seeing themselves in a relationship with someone unable to appreciate their input. Research participants expressed regrets about investing much effort in their families yet being treated as “nothing or nobody.” Reflecting on their marriage, they realized they were not appreciated or valued, despite “doing everything for the family.” They saw their partners using them as a tool to gain certain benefits (e.g., to “tidy the house, because you are a maid, as a hostess”). From another side, individuals reported seeing themselves as directly involved in maintaining their marriage’s dysfunctional structure. Individuals accepted that their passive behavior influenced marital problems or even led to the relationship’s collapse. For some participants, this realization with much pain and suffering.

*When [my eyes] opened, it was terribly painful, it hurt a lot, it hurt for months. When I found out about my codependence, I don't know, maybe for a month I was in such a shock. (Paul, 48)*

#### Accepting the Ongoing

Some divorcees reported working on accepting the past and present. For them, it seemed to be the only way to work through the issues they could not change. People encouraged themselves that everything would be resolved, and all came back to a particular normal. Karen (47), 1 year into divorce, argued, “You try to let go, analyze the situation, the why, and then you realize that you cannot change the fact; you have to accept it.” Somewhat similar to the acceptance is what divorcees expressed as letting the divorce process go parallel to their everyday lives. They argued that heightened emotions related to divorce took over and “sucked them” initially. However, with time, especially when the legal process takes years, divorcees had to let the legal process “go like something natural, which is happening along with all your other things” (Virginia, 39, 4 years into divorce).

## Discussion

This qualitative study was conducted to explore the change of the self in people going through a lasting divorce process. According to the present study, people experienced a self-disruption amidst an ongoing divorce, particularly related to spousal and parental roles. Individuals lost the sense of self-continuity over time, thinking that important parts of the self they had built in the past were lost and that the future self to build on vaguely existed or was continuously interrupted. Consistent with the two-dimensional change model (Mattingly et al., 2014), these results could be explained through self-contraction. Because the self is formed and apprehended through everyday practice with family members, the inability to interact freely and uncontrollably with them and enact the “traditional” roles of a parent or spouse creates a clash with the self, followed by feelings of self-related loss, pain and stress (Balmer et al., 2018; Mattingly et al., 2014).

Despite the results’ initial straightforwardness, the losses are more complicated. Losses of a spousal role were largely connected to the loss of future-oriented togetherness dreams. Parental role losses also associated with the inability to enact parental responsibilities, the idea of what it meant to be a “traditional” parent and behave according to it. Particularly, divorcees who could not freely interact with their children felt these changes. Consequently, results show that disconnection with ideas and dreams connected to the closest people plays an essential role when discussing divorce-related losses. Marriage and being in a traditional family remain highly valued aspects for many people (Hopper, 2001). This notion is particularly prevalent among Lithuanians viewing family life, including raising children as the center and foremost priority of a person's life (Kanopienė et al., 2015). Therefore, divorce becomes highly stressful because it threatens highly valued core self-aspects connected to the notion of being a part of the traditional family structure.

Next to the losses of valued self-aspects, some losses provided the opportunity for individuals to experience rebuilding a more valued self. These changes associated with increased positivity and a sense of self-liberation. Scholars underline that although individuals usually strive for positive self-expansion, they also add negative parts or neglect more valued self-parts (Mattingly et al., 2014). As people lose negatively experienced self-parts, they move closer to an ideal self and consequently experience increased well-being (Lewandowski & Bizzoco, 2007). However, creating a more valued self was not without challenges. Divorcees experienced the self as stuck, “hanged in the process,” and therefore, restricted in various ways that conveyed feelings of the impossibility of a definite self-reconstruction. The role-relational ambiguity phenomenon comes closest to explaining this experience (Tabor, 2019). Results of qualitative research, which focused on individuals living apart from their spouses without the intent to divorce, showed that finding the self in a lasting ambiguous situation without a clear understanding of what roles an individual is supposed to perform and what is expected of them associated with much negatively experienced self and situation unclarity (Crabtree & Harris, 2020).

The constant alertness and insecurity about being unfairly attacked, blamed, and coerced strengthened the above experiences. Next to attacks from the ex-spouse, ongoing litigation, and interactions with institutions complicated the divorce and timely self-redefinition. Despite associated professionals’ duty to provide support, divorcing parents often felt unheard, misunderstood, or misrepresented, questioning their role and effectiveness. Scholars underline that when divorcees lose trust, they are reluctant to cooperate with officials (Bouma et al., 2020). In this way, associated professionals lose the possibility to influence divorce-related conflict, indirectly and directly restricting effective and timely self-transition. Consequently, we argue that the stress of long-term divorce is not only related to adverse losses and emotional work to adjust for that. It also comes from the continuity of the process without a clear end line and it results in the perceived hostile environment enforcing the experiences of the self as incomplete, helpless, attacked, and stuck, unable to timely, and according to their own wishes, proceed with the process and finalize it.

Our results indicated that divorcees use particular strategies to counteract the negative experiences mentioned above, meaning that they see themselves as individuals actively engaging in the inner work to support self-transformation. Counteracting the blame and underlining being strong and truthful individuals who act according to moral standards can increase the sense of agency and self-coherence, while reconstructing the past and the past-self can help provide further motivation to move closer to a more valued self. According to the literature, people employ various strategies to protect, preserve, and promote their inner selves, which helps them to become stronger (Alicke & Sedikides, 2009; Hepper et al., 2010). However, the strategies can be efficient or inefficient depending on the situation and the amount of use. For example, behaving in a morally correct way or seeing themselves as strong and rightful individuals provided more strength to divorcees. However, the literature indicates that employing this strategy could lead to developing moral superiority, undermining others’ experiences, or showing a tendency to present the self as more moral and better than one is (Dong et al., 2019).

We could argue similarly about the acceptance strategy. Redefining identity through accepting divorce-related experiences as a part of the self had helped participants to manage their emotional reactions and to continue with their lives. Literature underlines that more self-accepting individuals show less need to prove themselves to others and they tend to be more objective in their self-evaluations (Chamberlain & Haaga, 2001). This strategy could associate with positively experienced self-redefinition and clarity. However, being sure of oneself and one’s ways of dealing with the situation could hamper accepting others’ perspectives and preventing timely conflict de-escalation. Therefore, as people generally use strategies for self-enhancement and protection purposes, they do not always support effective conflict dissolution and consequently, self-redefinition.

### The Study’s Strengths and Limitations

By allowing participants to reflect on their experiences in real time, results point toward three interconnected main categories explaining self-transformations during the lasting marital dissolution process. Overall, these categories indicate the ongoing transitional self-redefinition process. This process starts mainly with disruptions in spousal and parental roles and it continues through self-transition, which requires internal support strategies to counteract perceived restrictions.

This study is not without certain limitations. This sample was self-selected, relatively socioeconomically advantaged, and better educated. Limitations around received, rehearsed, and edited narratives should also be considered because several participants in this group appeared to be well-informed about lay and psychological theories regarding psychological violence, co-dependency, and parental alienation. Furthermore, this is a qualitative study, thus, we are not claiming that the findings can be generalized simplistically; they should be treated as suggestions for issues worthy of additional investigation rather than as definitive statements.

### Theoretical and Practical Implications

Specific areas that might be interesting and beneficial for future studies stood out during the research process. Notably, the so-called moral-hypocrisy phenomenon (Dong et al., 2019), re-partnering, and parent–child relationships from the parent perspective could play a substantial role in the ongoing conflicts. Furthermore, despite the prevalence of qualitative studies investigating the self phenomenon, employing a quantitative approach provides a richer and more profound understanding of divorcees’ contextualized experiences. Last, we cannot emphasize more the importance of early interventions to support people through the first years of an emotionally intensive decoupling process. Associated professionals should remember that the stress of long-term divorce is not only related to perceived losses and emotional work to adjust for that. It also comes from the continuity of the process without a clear end line and results in a perceived hostile environment. Providing trust and safety should be the main aspects of tackling uncertainties and insecurities. Considering emotional labor is significantly high for individuals involved in litigation processes, lawyers could become individuals who help divorcees prepare psychologically for the legal process and prevent overwhelming reactions to triggers.
